# KDM2A Targets PFKFB3 for Ubiquitylation to Inhibit the Proliferation and Angiogenesis of Multiple Myeloma Cells

**DOI:** 10.3389/fonc.2021.653788

**Published:** 2021-05-17

**Authors:** Xinling Liu, Jiaqiu Li, Zhanju Wang, Jie Meng, Aihong Wang, Xiaofei Zhao, Qilu Xu, Zhen Cai, Zhenbo Hu

**Affiliations:** ^1^ Department of Hematology, Laboratory for Stem Cell and Regenerative Medicine, Clinical Research Center, Affiliated Hospital of Weifang Medical University, Weifang, China; ^2^ Department of Oncology, Clinical Research Center, Affiliated Hospital of Weifang Medical University, Weifang, China; ^3^ Department of Dermatology, Weifang Hospital of Traditional Chinese Medicine, Weifang, China; ^4^ Department of Hematology, The First Affiliated Hospital, Weifang Medical University, Weifang, China; ^5^ Department of Hematology, The First Affiliated Hospital, School of Medicine, Zhejiang University, Hangzhou, China

**Keywords:** KDM2A, PFKFB3, ubiquitination, multiple myeloma, proliferation

## Abstract

The lysine demethylase KDM2A (also known as JHDM1A or FBXL11) demethylates histone H3 at lysine K36 which lead to epigenetic regulation of cell proliferation and tumorigenesis. However, many biological processes are mediated by KDM2A independently by its histone demethylation activity. In the present study, we aimed to characterize the functional significance of KDM2A in multiple myeloma (MM) disease progression. Specifically, we defined that one of the key enzymes of glycolysis PFKFB3 (6-phosphofructo-2-kinase) is ubiquitylated by KDM2A which suppresses MM cell proliferation. Previous study showed that KDM2A and PFKFB3 promoted angiogenesis in various tumor cells. We further reveal that KDM2A targets PFKFB3 for ubiquitination and degradation to inhibit angiogenesis. Several angiogenic cytokines are also downregulated in MM. Clinically, MM patients with low KDM2A and high PFKFB3 levels have shown worse prognosis. These results reveal a novel function of KDM2A through ubiquitin ligase activity by targeting PFKFB3 to induce proliferation, glycolysis and angiogenesis in MM cells. The data provides a new potential mechanism and strategy for MM treatment.

## Introduction

The pathogenic role of histone modification leads to various cancers (1). In the nucleosome, DNA is packaged by two sets of core histones, H2A, H2B, H3 and H4, whose amino acid residues can be regulated by diverse post-translational modifications such as methylation, acetylation or phosphorylation (2). The lysine demethylase KDM2A, also known as JHDM1A, FBXL11 or Ndy2, demethylates histone H3 at lysine K36 (3). Besides, KDM2A has been demonstrated to demethylate lysines of non-histone proteins such as the NF-kappaB, p65 subunit or beta-catenin (4, 5). KDM2A also stimulates ubiquitination and stabilizes p53-binding protein 1 (53BP1), promotes recruitment of 53BP1 to DNA breaks, and finally leads to increased DNA damage-induced genomic instability (6). Another lysine demethylase KDM2B, structurally similar to KDM2A, also exhibits an E3 ubiquitin ligase activity (7). Therefore, the range of KDM2A substrates might extend beyond histones. In addition, based on different context, KDM2A exhibits proliferative/antiproliferative properties in various cancers (8–11). However, the function of KDM2A in MM is rarely reported and understood.

Cancer cells prefer glycolysis as the major source of energy to maintain their proliferation. The 6-phosphofructo-2-kinase/fructose-2,6-biphosphatase 3 (PFKFB3) plays a crucial role to boost glycolysis (12). Recently, PFKFB3 is demonstrated increasingly to be involved in non-glycolysis-dependent biological functions, such as angiogenesis (13), cell cycle regulation (14), and autophagy (15). PFKFB3 is frequently overexpressed in diverse cancers and stimulated by oncogenic signaling. As reported, PFKFB3 can be modified in various ways. For example, phosphorylation of PFKFB3 at Ser461 by AMPK increases glycolytic flux and promotes cancer cell proliferation (16). Carbon monoxide (CO) reduces methylation of PFKFB3, which causes metabolic rewiring toward the pentose phosphate pathway (PPP) and acquisition of chemoresistance. Ubiquitin-mediated proteasomal degradation of PFKFB3 protein also reroutes glycolysis to the PPP (17). Previously, we found PFKFB3 was overexpressed in MM (18). As thus, the rationale of this study is to understand if the role of KDM2A played in regulating proliferation of MM cells is through PFKFB3 ubiquitylation in order to learn underlining mechanism and develop new strategy for MM treatment. Here, we show that KDM2A induced ubiquitylation of glycolytic enzyme PFKFB3 and negatively regulated MM cell proliferation and angiogenesis.

## Materials and Methods

### Cell Culture and Reagents

MM cell lines RPMI8226 and MM.1S were purchased from DSMZ(Braunschweig, Germany), and CAG and ARP1 were generously provided Dr. Qing Yi (Center for Hematologic Malignancy, Research Institute, Houston, TX, USA). All the cell lines were maintained in RPMI-1640 medium (Corning Cellgro, USA) supplemented with 10% fetal bovine serum (FBS) (GIBCO, CA, USA), 100U/ml penicillin and 100μg/ml streptomycin at 37°C and 5% CO_2_ in air. BM samples from MM patients and peripheral blood mononuclear cells (PBMCs) from healthy donors were obtained after informed consent was provided following approval by the Ethics Committee of Affiliated Hospital of Weifang Medical University. MG132 and Cycloheximide were purchased from Sigma-Aldrich. Primary antibodies against KDM2A, PFKFB3, CDK6, MCL-1 and ubiquitin were purchased from Abcam (Cambridge, UK). Anti-VEGF antibody was purchased from Santa Cruz Biotechnology (Santa Cruz, CA, USA). GAPDH and Bax were purchased from Cell Signaling Technology (Danvers, USA). HA was obtained from Roche (Basel, Switzerland). Horseradish peroxidase (HRP)-conjugated anti-rabbit antibodies were obtained from Jackson ImmunoResearch Laboratories (Lancaster, USA). Lipofectamine 2000 reagent (Invitrogen) was used for transient transfection. The shRNA or siRNA sequences used were shown in **Supplementary Table S1**.

### Cell Proliferation Assay

A cell counting kit-8 (CCK-8) assay kit (Dojindo, Japan) was used to measure MM cell proliferation and viability. MM cells (1×10^5^/100 μl/well) were seeded in 96-well plates and treated with 10μl of CCK-8 solution co-incubating at 37°C for 2 h. Then, absorbance was measured at 450 nm using an enzyme microplate reader. Cell viability (%) = OD value of the test sample/OD value of the control×100.

### Stable Cell Line Generation

For stable KDM2A knockdown, MM cells were transfected with green fluorescent protein-containing (GFP) shRNA lentiviral particles directed against human KDM2A or with empty vector. Cells were selected in culture media containing puromycin (2μg/ml) for 2 weeks.

### ELISA

Human VEGF and IL-32 was determined by ELISA (R&D Systems) according to the manufacturer’s instructions.

### Glucose Uptake and Lactate Production Measurements

The assays were carried out according to the protocol of glucose uptake assay kit (colorimetric, Abcam) and lactate Assay Kit (Cayman Chemical).

### Tube Formation Assays

2×10^5^ cells were seeded on 24-well plates coated with BD Matrigel™ Matrix and incubated in indicated medium for 24 h at 37°C. The formation of capillary-like structures was observed and captured under a light microscope. The total length of tubes was analyzed by ImageJ.

### Xenograft Tumor Studies

CAG cells (5×10^6^) with KDM2A overexpression resuspended in 50μl RPMI-1640 were injected subcutaneously into 4-week-old male NOD-SCID (non-obese diabetic-severe combined immunodeficient) mice. The experiments were terminated 3 weeks after cell injection, and tumors were weighed. Tumor volume was calculated according to the following formula: V= 4π/3 × (a/2)^2^ × b/2, where a is the tumor width and b is the tumor length. The mice were sacrificed when the tumor volumes reached approximately 3000 mm^3^. All animal experiments were carried out in accordance with the protocols of the Animal Ethics Committee of the Affiliated Hospital of Weifang Medical University.

### Co-IP and Western Blot

Whole-cell extracts were prepared in RIPA buffer (Cell Signaling Technology) with multiple protease inhibitors (Sigma-Aldrich) for 30 min. Total protein was pre-cleared with Protein A/G beads which were incubated with rotation for 2 h at 4°C. After centrifugation, the supernatant was moved to a new tube and the beads conjugated with specific antibody were added to the supernatant. The tube was rotated for 2 h at 4°C again. Then the tube was placed into a magnetic stand to collect the beads, and the supernatant was discarded. The beads were washed three times with wash buffer, and the complexes were eluted from the beads for sodium dodecyl sulfate–polyacrylamide gel electrophoresis (SDS–PAGE) and used for further analysis. Western blot was performed as described previously (18).

### Immunohistochemistry Analyses

Paraformaldehyde-fixed, Triton X-100-permeabilized cells from BM biopsy tissues of MM patients were used for immunofluorescence staining with specific antibodies to analyze the expression of KDM2A and PFKFB3. The experiment was approved by the Affiliated Hospital of Weifang Medical University Ethics Committees. Immunostaining scores were independently evaluated by two researchers who were blinded to the patients and samples. The percent of positive staining cells (< 50%, 50–70%, and > 70%) and the staining intensity were defined as following: absent (−), scarce (±), moderate (+) or strong (++). The expression levels of KDM2A and PFKFB3 were scored as follows: positive: > 70% positivity with scarce (±) to strong(++) staining or 50–70% positivity with moderate (+) to strong (++) staining; weakly positive: 50–70% positivity with scarce (±) staining or < 50% positivity with scarce (±) to strong (++) staining; and negative: < 50% positivity with scarce (±) to absent (−) staining. The high expression group composed of positive cases, whereas the low expression group contained weakly positive and negative cases as defined above. The average sum of integrated optical density (IOD) of each sample was calculated using ImageJ software.

### Sample Preparation for MS and LC-ESIMS/MS Analysis by Q Exactive HF

The purified protein samples prepared from IP were separated by SDS-PAGE and subjected to LC-ESIMS/MS analysis which was performed by Micrometer Biotech Company (Hangzhou, China).

### Statistical Analysis

All data were presented as the mean ± standard deviation (S.D.). All analyses were conducted using GraphPad Prism 6.0 (GraphPad Software, San Diego, CA, USA). Two-tailed Student’s t-test was used to estimate significant differences between two groups. P*<0.05 was considered to be significant. All experiments were performed in triplicate as three independent assays.

## Results

### PFKFB3 Is a Substrate of KDM2A

**Figure 1:**
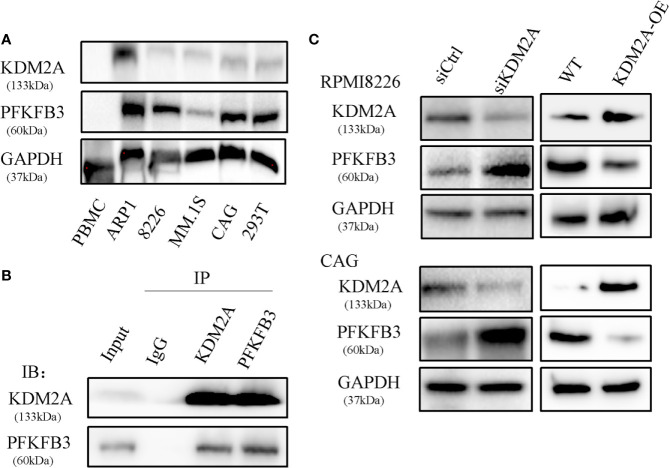
PFKFB3 is a substrate of KDM2A. **(A)** Western blot analysis of expression of PFKFB3 and KDM2A in MM cell lines, PBMCs and 293T cells. PBMC: normal healthy donors’ peripheral blood mononuclear cells as the control. **(B)** Co-IP assay to detect that endogenous KDM2A and PFKFB3 interacted with each other in RPMI8226 cells. **(C)** Western blot analysis showed that PFKFB3 protein levels increased in RPMI8226 and CAG cells transfected with siKDM2A. The gain-of-function assays validated the suppressive effect of KDM2A on PFKFB3 as well. GAPDH was used as a loading control. All the experiments were repeated three times.

Our previous study has demonstrated that metabolic enzyme PFKFB3 promoted MM cell proliferation. By mass spectrometry analysis, we demonstrated that PFKFB3 may be ubiquitylated and identified KDM2A as a new binding target. The representative spectra of KDM2A and PFKFB3 are shown in **Supplementary Figure 1A**. Firstly, the expression of KDM2A and PFKFB3 was detected by western blotting in MM cell lines, PBMCs and 293T cells. PFKFB3 protein showed obviously high expression in MM cells except of relatively low expression in MM.1S. KDM2A protein had relatively low expression level (**Figure 1A**). To further explore the underlying mechanism of PFKFB3 oncogenic function, the interaction between PFKFB3 and KDM2A was determined by co-immunoprecipitation (Co-IP) assay in RPMI8226 cells (**Figure 1B**). Furthermore, Downregulation of KDM2A gene expression using small interfering RNA (siRNA) increased PFKFB3 protein expression level in RPMI8226 and CAG cell lines. Therefore, the gain-of-function assays validated the suppressive effect of KDM2A on PFKFB3 in MM cells (**Figure 1C**). Taken together, our results provide evidence that PFKFB3 is a direct target of KDM2A.

### KDM2A Promotes MM Cell Proliferation and Cell Cycle Progression

**Figure 2:**
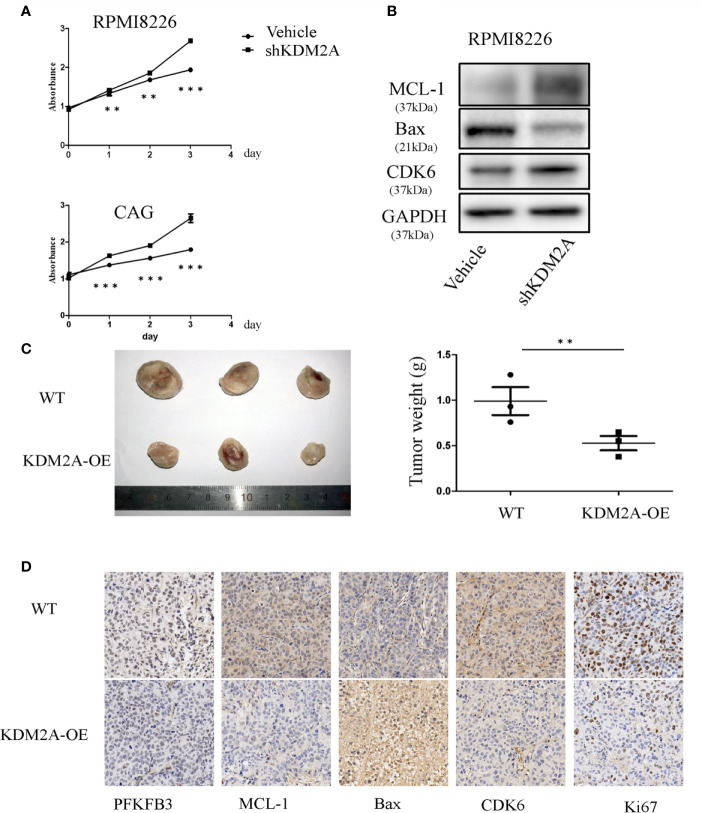
KDM2A mediates MM cell proliferation. **(A)** Proliferation of RPMI8226 and CAG cells was determined by CCK-8 assay after shKDM2A transfection. The results are representative of three independent experiments. **(B)** Knocking down of KDM2A decreased the expression of Bax and increased MCL-1 and CDK6 in MM cells by Western blot analysis. GAPDH was used as loading control. **(C)** Photographs of tumor masses were shown on the left panel and the tumor weight was measured and shown on the right panel after excised 3 weeks inoculation of CAG cells with KDM2A-WT or KDM2A-OE into NOD-SCID mice. **(D)** Immunohistochemistry staining for tumor tissue from KDM2A-WT and KDM2A-OE NOD-SCID Mice. (*P < 0.05, **P < 0.01, ***P < 0.005, data were represented as mean ± SD).

To learn if the biological functions of KDM2A and PFKFB3 in MM cells are opposite, we measured the effect of KDM2A on MM cell proliferation *in vitro*. The results showed that knockdown of KDM2A using small hairpin RNA (shRNA) promoted cell proliferation (**Figure 2A**). Due to promoting cell cycle progression as a non-canonical function of PFKFB3, we observed that in MM cells shKDM2A increased CDK6 protein level, which was involved in the core cell cycle that drove cell proliferation (19, 20). Moreover, myeloid cell leukemia-1 (MCL-1) as an anti-apoptotic protein was increased and Bax as pro-apoptotic protein was decreased after KDM2A knockdown (**Figure 2B**). Consistently, overexpression of KDM2A inhibited myeloma tumor growth *in vivo* (**Figures 2C, D**). Collectively, these data suggest that shKDM2A increases MM cell proliferation.

### shKDM2A Promotes Glycolysis Activity and Tube Formation of HUVECs

**Figure 3:**
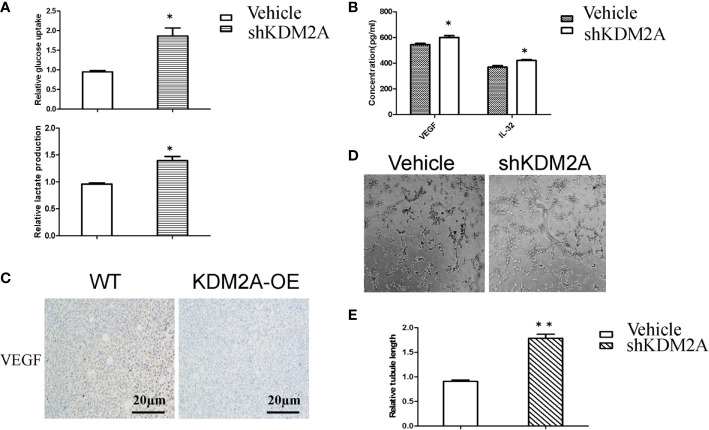
shKDM2A promotes glycolysis activity and tube formation of HUVECs. **(A)** Glucose uptake and lactate production were detected in MM cells with shKDM2A as described in the Material and Methods using a glucose uptake assay kit and a lactate assay Kit.*P < 0.05. **(B)** ELISA assays showed that shKDM2A MM cells increased secretion of VEGF and IL-32 in culture medium. *P<0.05. **(C)** Representative IHC images for VEGF in the mice were shown (scale bar, 20 µm). **(D)** MM cells with shKDM2A culture medium enhanced tube formation ability in HUVECs seeded on Matrigel. Images were taken using a light microscope (×100). **(E)** The quantitative data of the tube formation assays. Quantitative data were from 3 independent experiments. *P < 0.05. The above representative results were verified in RPMI8226 cell lines.

AMP-activated protein kinase (AMPK) maintains energy homeostasis, which can positively regulate PFKFB3 to promote glycolysis, thereby increasing glucose uptake for cancer cell proliferation (21). A study reported that inhibiting mTOR finally reduced PFKFB3 expression to negatively regulate angiogenesis and cell migration (22). It has been widely confirmed that PFKFB3 mediated tumor growth and angiogenesis (23, 24). In order to detect whether shKDM2A plays a similar role in glycolysis as that of PFKFB3, we determined the levels of glucose uptake and lactate production in MM cells. As shown in **Figure 3A**, glucose uptake and lactate production were both increased in MM cells, which means glycolysis enhanced with KDM2A knockdown. In addition, vascular endothelial growth factor (VEGF) is a key component of pro-angiogenic activity, and our previous study also demonstrated that IL-32 promoted angiogenesis. In consideration of high expression of IL-32 in multiple myeloma (25, 26), we then measured the levels of VEGF and IL-32 in MM cells by ELISA. As expected, the median levels of VEGF and IL-32 were higher in the shKDM2A group than in the control group (**Figure 3B**). Meanwhile, immunohistochemical analysis showed that KDM2A-OE tumors exhibited more negative staining for VEGF than control group (**Figure 3C**). Based on above data, tube formation assay further revealed that KDM2A knockdown promoted the formation of capillary-like structures in HUVECs (**Figures 3D, E**). These data indicate that shKDM2A promotes glycolysis activity and tube formation of HUVECs.

### KDM2A Induces Instability of PFKFB3

**Figure 4:**
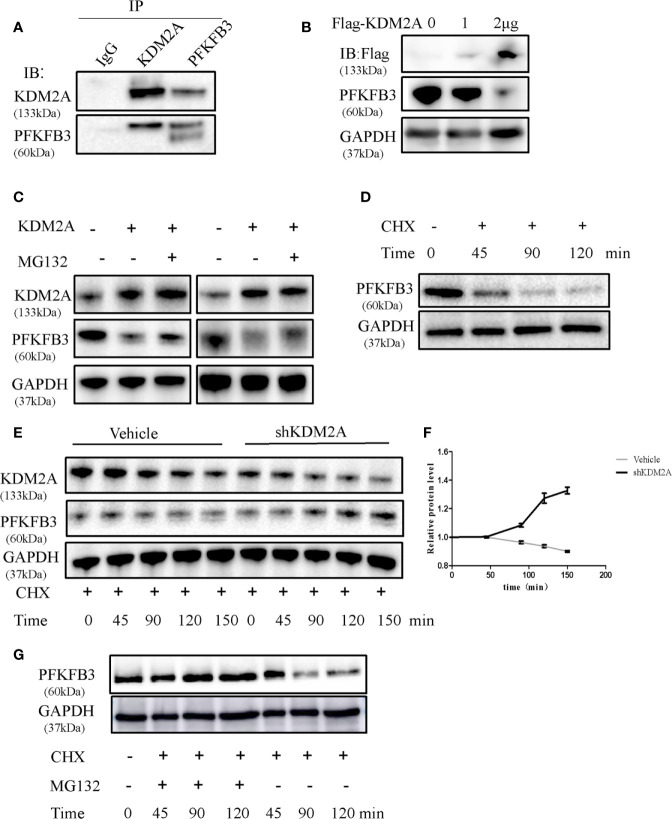
KDM2A induces instability of PFKFB3. **(A)** Endogenous KDM2A and PFKFB3 interacted with each other in 293T cells, which was determined by Co-IP assay and Western blot. **(B)** Dose-dependent reduction of PFKFB3 in 293T cells transfected with KDM2A expression vector. GAPDH was used as loading control. **(C)** KDM2A induced ubiquitin proteasome system of PFKFB3. MM cells with KDM2A overexpression were treated with MG132 for 2 hours. PFKFB3 protein expression was evaluated as determined by Western blotting. **(D)** PFKFB3 protein degradation in MM cells treated with CHX in a time series. **(E)** Comparison of PFKFB3 protein degradation between the wild-type KDM2A and shKDM2A in cells with a time-course CHX treatment. **(F)** Quantification of **(E)** in triplicate. **(G)** MG132 inhibited the degradation of PFKFB3 protein detected by western blot analysis.

As shown in **Figure 1C**, KDM2A negatively regulated endogenous PFKFB3 protein level. We next examined whether KDM2A regulates the stability of PFKFB3 protein. Firstly, we performed Co-IP assay in HEK293T to detect the exogenous interaction of KDM2A and PFKFB3 (**Figure 4A**). Then we overexpressed Flag-tagged KDM2A in 293T cells which showed dramatically decrease PFKFB3 protein level in a dose-dependent manner (**Figure 4B**). Treatment of cells with MG132, a 26S proteasome inhibitor, effectively blocked PFKFB3 degradation (**Figure 4C**), confirming that PFKFB3 is degraded by KDM2A through the proteasome pathway. Furthermore, we treated cells with cycloheximide (CHX) to measure the half-life of PFKFB3. The knockdown of KDM2A decreased the degradation of PFKFB3 compared with the control vector (**Figures 4D-F**). Meanwhile, to determine whether the proteasome pathway is necessary for regulating of PFKFB3 protein stability, we treated the cells with CHX or MG132 for 120min and determined the PFKFB3 protein level by Western blotting. The result showed that MG132 inhibited the degradation of PFKFB3 protein (**Figure 4G**) suggesting proteasome pathway was involved in PFKFB3 protein degradation.

### Interaction Between KDM2A and PFKFB3

**Figure 5:**
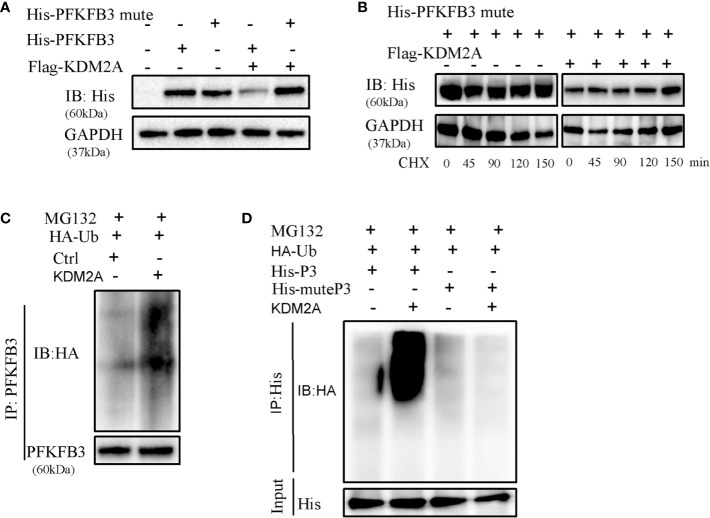
Determination of interaction between KDM2A and PFKFB3. **(A)** Detection of the effect of KDM2A on PFKFB3 or PFKFB3 mutant degradation in 293T cells. **(B)** The turnover rate of PFKFB3 mutant was not changed in response to KDM2A overexpression with CHX treatment. **(C)** KDM2A generated ubiquitylation on PFKFB3. **(D)** KDM2A overexpression induced strong ubiquitylation of PFKFB3, but not on the PFKFB3 mutant protein.

The above Co-IP assay results showed that endogenous or exogenous KDM2A and PFKFB3 were bounded each other. Furthermore, we established PFKFB3 mutant with deletion of VLVIC 383-387, which is a potential SUMO-interacting motifs. The process of SUMOylation is putting a small ubiquitinlike modifier (SUMO) to protein substrates at specific lysine site (27, 28). Then we observed that KDM2A did not induce the degradation of PFKFB3 mutant (**Figure 5A**). Consistently, KDM2A overexpression did not change the stability of PFKFB3 mutant (**Figure 5B**), suggesting that the latter was resistant to KDM2A. As shown in **Figure 5C**, KDM2A strongly enhanced the ubiquitylation of PFKFB3. Moreover, KDM2A induced a dramatic enhancement in the ubiquitylation of PFKFB3, but not of the PFKFB3 mutant (**Figure 5D**). In summary, KDM2A interacts specifically with PFKFB3 to induce its degradation by ubiquitination pathway.

### Protein Levels of KDM2A and PFKFB3 Are Negatively Correlated in Multiple Myeloma Patients

**Figure 6:**
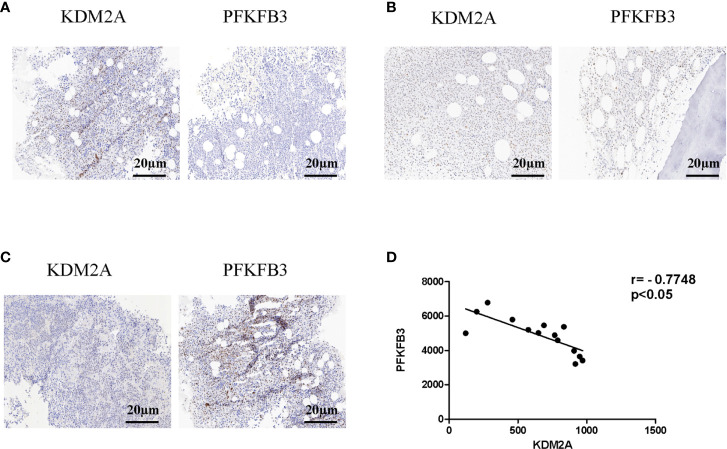
Protein levels of KDM2A and PFKFB3 are negatively correlated in multiple myeloma patients. **(A-C)** Representative images of staining with KDM2A antibodies in MM patients’ BM tissues showed strong **(A)**, moderate **(B)**, and weak **(C)** expression, respectively, and the expression of PFKFB3 is just the opposite of KDM2A (scale bar, 20 µm). **(D)** Pearson correlative analysis of semi-quantitative IOD values for KDM2A and PFKFB3 indicates that protein levels of KDM2A and PFKFB3 are negatively correlated in MM patients.

To further analyze the relationship between KDM2A and PFKFB3, bone marrow (BM) samples (n = 14) from MM patients were analyzed by immunohistochemistry. Statistical analysis showed that with the severity of the disease, KDM2A protein levels decreased (**Figures 6A-C**). Meanwhile, BM tissue with high expression of KDM2A exhibited a low expression of PFKFB3 protein, and vice versa. In order to ascertain the correlation of KDM2A and PFKFB3 expression, Pearson correlation test was performed based on IOD values. The Pearson correlation coefficient of KDM2A and PFKFB3 was -0.77 (**Figure 6D**), indicating that the expression of the two proteins was negatively correlated. Consistent with these data, we found that the KDM2A and PFKFB3 protein levels were significantly correlated with the ISS (International Staging System) stage of multiple myeloma, but not with other clinicopathological parameters (**Table 1**), which indicated that KDM2A and PFKFB3 may play a role in MM progression.

**Table 1:** Analysis of correlation between KDM2A or PFKFB3 protein levels and clinicopathological parameters of multiple myeloma patients.

	KDM2A expression				PFKFB3 expression			
Variable	n	Low	High	p-Value	n	Low	High	p-Value
				0.481				0.052
Age								
≤60 years	10	7	3		10	2	8	
<60 years	4	2	2		4	3	1	
Gender				0.027				0.207
Female	11	8	3		11	3	1	
ISS				0.027				0.030
Stage I	5	1	4		5	4	1	
Stage II	4	3	1		5	4	1	
Stage III	5	5	0		5	1	4	
Durie-Salmon staging				0.543				0.543
Stage IIA	1	1	0		1	0	1	
Stage IIB	2	2	0		2	1	1	
Stage IIIA	9	5	4		9	4	5	
Stage IIIB	2	1	1		2	0	2	

## Discussion

Studies have shown that KDM2A as a lysine demethylase was highly overexpressed in many cancers, such as breast cancer (8, 29), gastric cancer (9), lung cancer (30) and hepatoma (31). In addition, it can be downregulated by tumor microenviroment (28). In the present study, we demonstrated that expression of KDM2A was basically low in multiple myeloma cells. KDM2A mainly demethylates histone H3K36, however, more and more studies demonstrate that KDM2A has non-histone targets (4, 5, 32). Our data demonstrates that in myeloma cells KDM2A interacts with PFKFB3 and mediates its ubiquitination-induced degradation. Moreover, KDM2A knockdown promotes MM tumorigenesis and progression.

PFKFB3 is one of the pivotal glycolytic enzymes which promotes cancer cell proliferation (33–35). Our previous studies indicate that PFKFB3 inhibition alleviated MM cell proliferation (18). In addition, the function of PFKFB3 is regulated by protein stability and transcriptional stimulation. For example, hypoxia stimulated PFKFB3 expression at the transcriptional level (36). PFKFB3 is subjected to proteosomal degradation through the E3 ubiquitin ligase APC/C-Cdh1 (37). LncRNA AGPG stabilizes PFKFB3 by preventing K302 ubiquitination (35). PFKFB3 activity is also regulated by post-translational modifications, such as phosphorylation (38), dimethylation (17) and acetylation (39). This study is the first to report PFKFB3 can be ubiquitylated by KDM2A in multiple myeloma. Meanwhile, previous studies reported that tumor angiogenesis can be promoted by PFKFB3-mediated glycolysis (24, 40), and the production of VEGF, the most powerful pro-angiogenic growth factor, is increased. Moreover, IL-32 as a pro-inflammatory factor has been demonstrated to promote angiogenesis (41). Our previous study showed that IL-32 was overexpressed in MM patients (42). In this study, we demonstrate that knockdown of KDM2A increases PFKFB3 level, which further increases IL-32 mRNA to enhance tube formation in HUVECs. Therefore, our finding is consistent with the other studies.

In summary, the present study demonstrates that the ubiquitination of PFKFB3 by KDM2A inhibits MM tumorigenesis. In other words, a decreased level of KDM2A in MM patients may indicate a poor clinical prognosis. Our findings further suggest that regulating KDM2A-mediated PFKFB3 ubiquitination may be a promising application in multiple myeloma treatment.

## Data availability statement

The raw data supporting the conclusions of this article will be made available by the authors, without undue reservation.

## Ethics Statement 

The studies involving human participants were reviewed and approved by Affiliated Hospital of Weifang Medical University. The patients/participants provided their written informed consent to participate in this study. The animal study was reviewed and approved by Affiliated Hospital of Weifang Medical University.

## Author Contributions 

XL designed and performed the experiments. JL performed experiments. ZW, JM, AW and XZ helped in experiments and analyzed data. ZW and QX collected primary samples for the study. XL, ZC and ZH supervised the study, wrote and revised the paper. All authors contributed to the article and approved the submitted version.

## Funding 

This study was supported by Projects of medical and health technology development program in Shandong province (Grant No. 2019WS601, 2013WS0291), Shandong Province Natural Science Foundation (Grants No. ZR2020QH096, ZR2020KC016), National Natural Science Foundation of China (Grant No. 81570157), Weifang Science and Technology Bureau (Grant No. 2020YQFK013).

## Conflict of Interest

The authors declare that the research was conducted in the absence of any commercial or financial relationships that could be construed as a potential conflict of interest.
