# Supplementary material for: KDM2A Targets PFKFB3 for Ubiquitylation to Inhibit the Proliferation and Angiogenesis of Multiple Myeloma Cells
Source: Front Oncol. 2021 May 17;11:653788. doi: 10.3389/fonc.2021.653788 (PMC8165180; doi:10.3389/fonc.2021.653788)
Supplement: Supplementary file 1 [file DataSheet_1.doc]

**Supplementary Data**

**A**

**
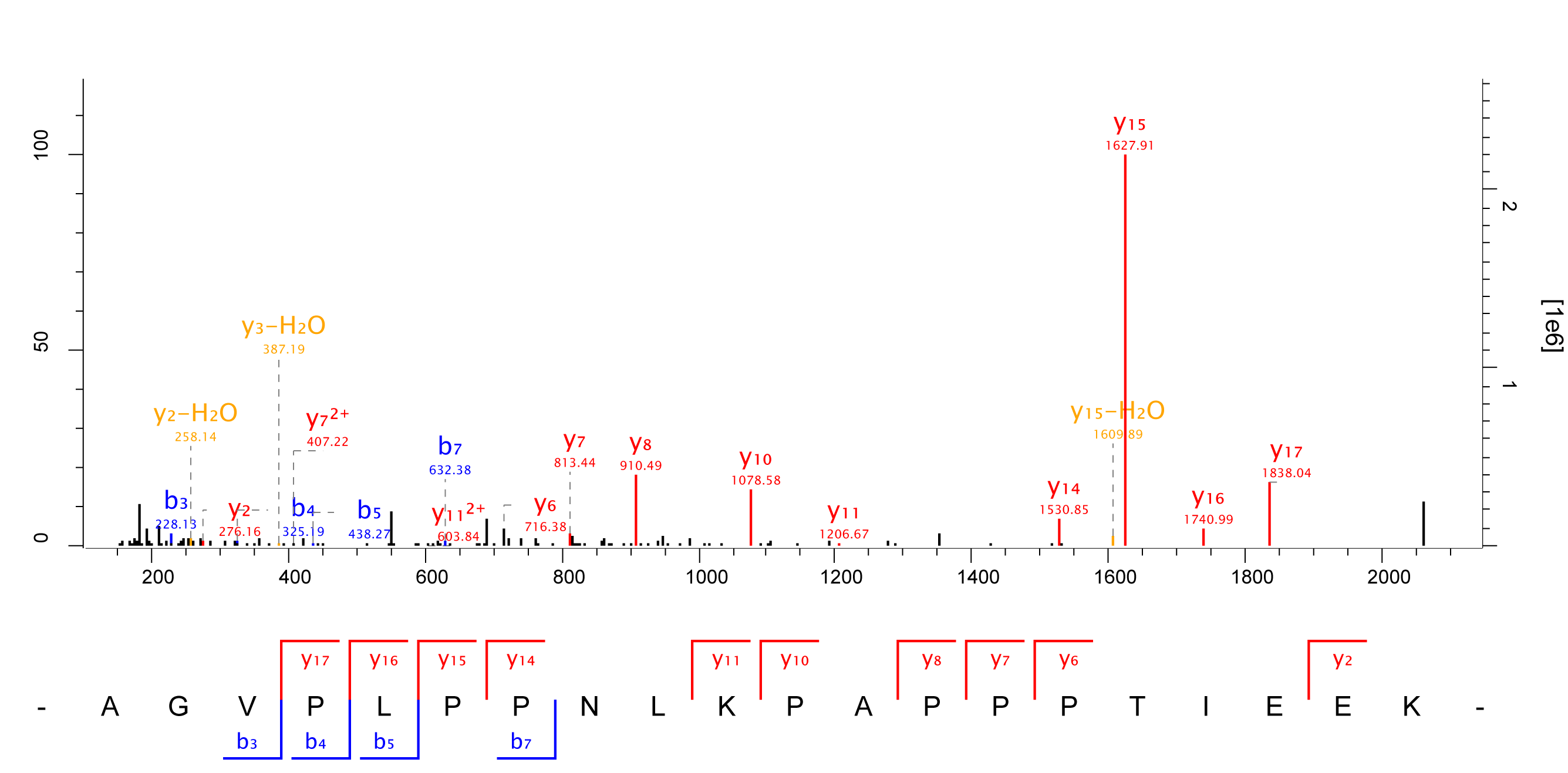

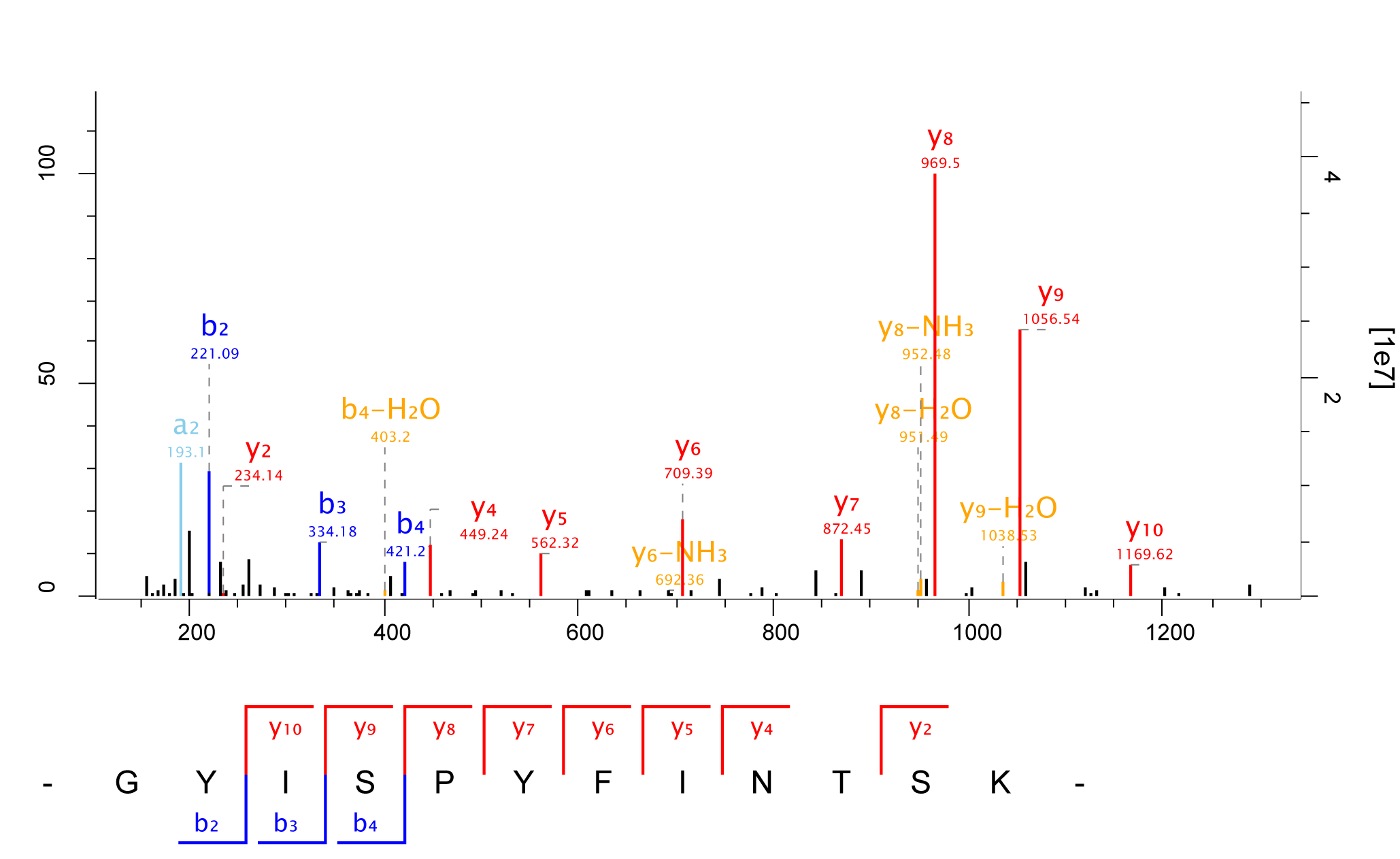
**

**
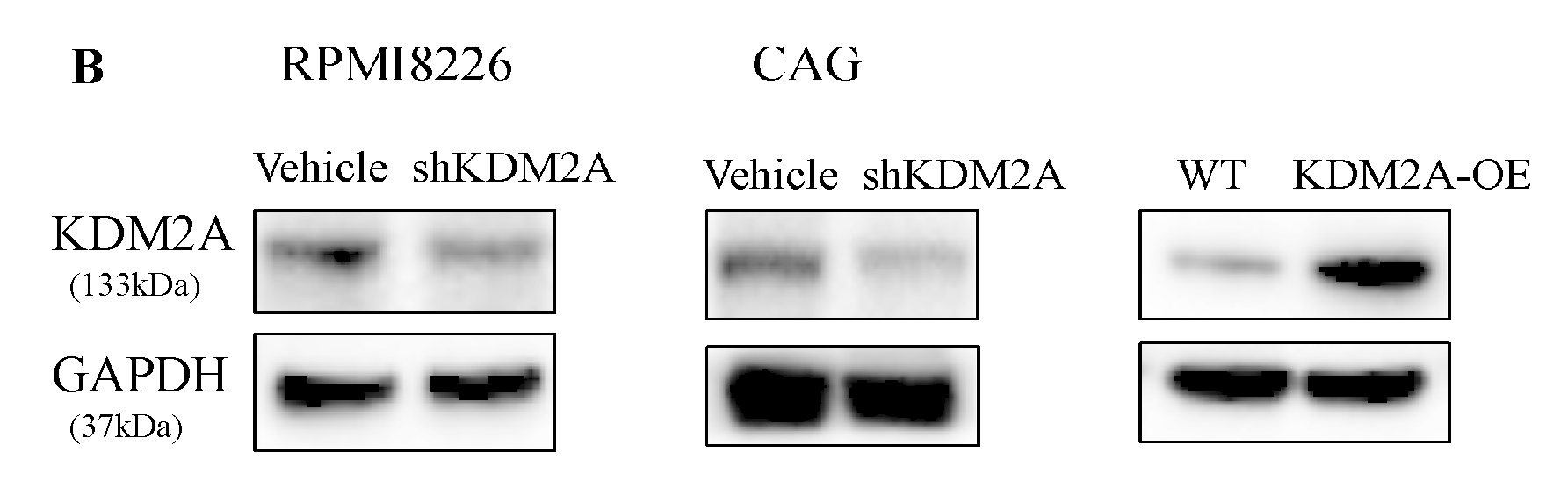
**

**Supplementary figure 1. A,** Mass spectrum of one unique peptide AGVPLPPNLKPAPPPTIEEK found in sp|KDM2A_HUMAN (left) and GYISPYFINTSK in PFKFB3 (right). **B,** KDM2A was stably knocked down or overexpressed in MM cells followed by western blotting.

**Table S1 Reagents used in our research**

| shRNA sequences | Sense (5'-3') | Anti-sense (5'-3') |
| --- | --- | --- |
| shRNA-Control | GTTCTCCGAACGTGTCACGTT | CAAGAGGCTTGCACAGTGCAA |
| shRNA-KDM2A | GCACACCAACAAATATAATGCT | CGTGTGGTTGTTTATATTACGA |
| Recombinant DNA | Source | Identifier |
| pCDNA3.1-Flag-KDM2A | Ribobio,Inc,In Guangzhou, China | N/A |
| pCDNA3.1-His-PFKFB3 MUT | Ribobio,Inc,In Guangzhou, China | N/A |
| pCDNA3.1-His-PFKFB3 | Ribobio,Inc,In Guangzhou, China | N/A |
| pCDNA3.1-HA-Ub | Ribobio,Inc,In Guangzhou, China | N/A |
